# Dysphagia‐Related Burden in Caregivers of Persons with Early‐Stage Dementia: Impact of Disease Stage and Diet Level

**DOI:** 10.1002/alz.088386

**Published:** 2025-01-09

**Authors:** Marissa Todd, Meredith Mackowicz‐Torres, Sara Gustafson, Samantha Shune, Ashwini Namasivayam‐MacDonald, Nicole M. Rogus‐Pulia

**Affiliations:** ^1^ University of Wisconsin‐Madison School of Medicine and Public Health, Madison, WI USA; ^2^ William S. Middleton Memorial Veterans Hospital, Madison, WI USA; ^3^ University of Oregon, Eugene, OR USA; ^4^ McMaster University, Hamilton, ON Canada

## Abstract

**Background:**

Caregiver burden, or the impact of caregiving, commonly occurs in caregivers of persons with dementia (PWD); however, prior research focused on caregiver burden in this population has not considered the impact of dysphagia. Therefore, the purpose of this study was to measure dysphagia‐specific burden in caregivers of PWD and examine its relationship to general caregiver burden, as well as the PWD’s current diet level and dementia severity.

**Method:**

Data were collected from PWD‐caregiver dyads participating in a prospective, dysphagia‐focused clinical trial at the initial study visit. The Clinical Dementia Rating (CDR) scale was administered to dyads to determine dementia severity. The Zarit Burden Interview (Zarit) and Caregiver Analysis of Reported Experiences with Swallowing Disorders (CARES) were administered to caregivers. Functional Oral Intake Scale (FOIS) scores were recorded for the PWD.

**Result:**

Twenty‐nine dyads were included. Caregivers were 93% female with a mean age of 65 years. PWD were 21% female with a mean age of 80 years. The mean CARES score was 2.86 (range = 0‐26; SD = 3.43) and the mean Zarit score was 28.1 (range = 5‐65; SD = 16.64). The median FOIS score for PWD was 7 (range = 5‐7). The highest CARES values were reported for the following experiences: being worried about the future, fear of their care recipient choking, and feeling “anxious.” A one‐way analysis of variance revealed no differences in CARES scores based on CDR stage. A Kendall tau rank correlation coefficient revealed a significant moderate, negative association between FOIS and CARES scores (r = ‐0.52; p<.002); while the moderate, positive association Pearson correlation between CARES and Zarit scores (r = .40; p = .03) was also significant.

**Conclusion:**

Findings revealed that dysphagia‐associated caregiver burden may trend similarly as general burden (Zarit) but is generally low in earlier stages of dementia. However, the more restrictive the PWD’s diet (lower FOIS scores), the greater the dysphagia‐related burden reported by caregivers. Higher ratings on specific CARES items can guide clinical decision‐making regarding support needed by caregivers of PWD with early‐stage disease. The relationship between dysphagia‐related burden and general burden may change with disease progression, warranting continued investigation.

